# Commissioning and operation status of the MAX IV 3 GeV storage ring vacuum system

**DOI:** 10.1107/S1600577521002599

**Published:** 2021-04-13

**Authors:** Marek Grabski, Eshraq Al-Dmour

**Affiliations:** a MAX IV Laboratory, PO Box 118, SE-221 00 Lund, Sweden

**Keywords:** vacuum, NEG coatings, synchrotron light source, MAX IV, thin films

## Abstract

The 3 GeV electron storage ring of the MAX IV laboratory is the first storage-ring-based synchrotron radiation facility with the inner surface of almost all the vacuum chambers along its circumference coated with non-evaporable getter (NEG) thin film. The coating provides a low dynamic outgassing rate and pumping of active gases. As the NEG coating was applied on an unprecedented scale, there were doubts concerning the storage ring performance. Fast conditioning of the vacuum system and over five years of reliable accelerator operation have demonstrated that the chosen design proved to be good and does not impose limits on the operation. The vacuum system performance is comparable with or better than that of other similar facilities around the world, where conventional designs were implemented. Observed pressure levels are low, and the electron beam lifetime is long and not limited by residual gas density. A summary of the vacuum performance is presented.

## Abstract 2

The 3 GeV electron storage ring of the MAX IV laboratory is the first light source storage ring that has the inner surface of nearly all the vacuum chambers coated with non-evaporable getter (NEG) thin film. The vacuum and the accelerator performance have proven successful.

## 1. Introduction   

### 1.1. MAX IV facility   

The MAX IV facility in Lund, Sweden, is composed of two storage rings with electron energies of 1.5 and 3 GeV. A linear accelerator (Linac) serves as the full energy injector to the two storage rings and as a driver for a short pulse facility. The nominal energy of the Linac is 3 GeV (Eriksson *et al.*, 2013 ▸). The MAX IV 3 GeV storage ring started commissioning in August 2015, and began to deliver photons to users in April 2017 (Tavares *et al.*, 2018 ▸).

### 1.2. The 3 GeV storage ring   

**Figure 1:**
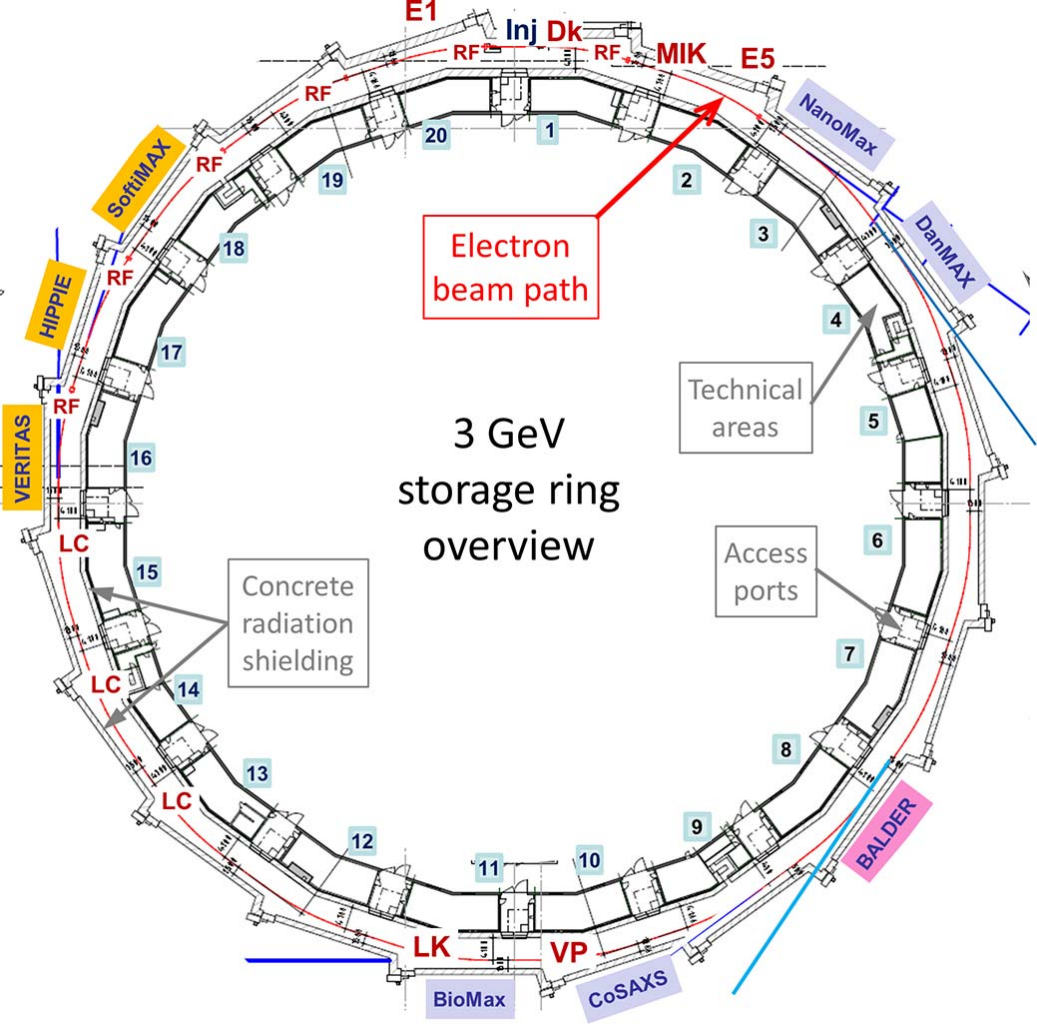
The layout of the 3 GeV storage ring at the MAX IV laboratory. Inj: injection straight; Dk: dipole kicker; MIK: multipole injection kicker; E1, E5: diagnostic beamlines for emittance measurement; RF: 100 MHz RF cavities; LC: 300 MHz Landau RF cavities; LK: longitudinal kicker cavity; VP: vertical pinger. Insertion devices in operation: in-vacuum undulators (IVUs) on NanoMax, DanMax, CoSAXS and BioMax beamlines, elliptically polarized undulators (EPU) on VERITAS, HIPPIE and SoftiMax; in-vacuum wiggler (IVW) on BALDER.

The 3 GeV storage ring is based on a multibend achromat (MBA) lattice. The large number of bending magnets was the main factor in obtaining ultralow horizontal emittance and ultimately achieving ultrahigh brightness, which was a requirement from the machine’s users (Einfeld *et al.*, 2014 ▸). The beam emittance is measured using two diagnostic beamlines, each with a different bending magnet as the source (Breunlin & Andersson, 2016 ▸). The storage ring has a 20-fold symmetry with a seven-bend achromat lattice and is 528 m in circumference. The general layout is depicted in Fig. 1 ▸.

In the storage ring there are six copper normal-conducting radio frequency (RF) cavities operating at 100 MHz and three Landau cavities (LC) operating at 300 MHz. The beam from the Linac is injected into the ring at full energy in the injection straight (Inj) of the long straight section of achromat 01.

**Figure 2:**
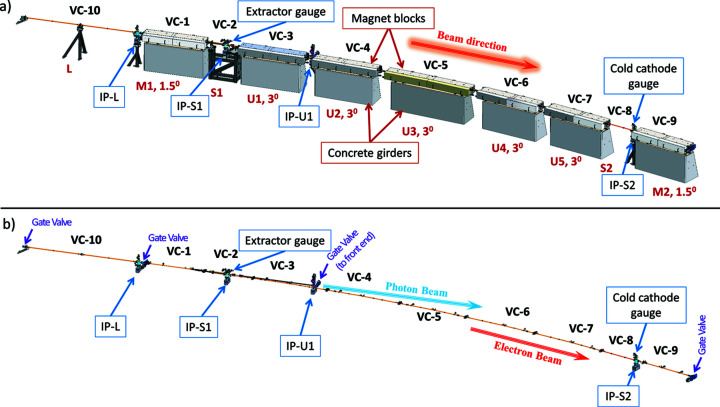
The standard achromat layout of the 3 GeV storage ring, (*a*) with magnet blocks, supports and concrete girders, and (*b*) without magnet blocks, supports and girders. L: long straight section; S1, S2: short straight sections 1 and 2; M1, M2: matching cell magnet blocks 1 and 2; U1–U5: unit cell magnet blocks; IP: ion pumps; VC-*n*: vacuum chamber types and vacuum gauge distribution and valves.

Each achromat has a total length of 26.4 m and contains seven magnet blocks of two types: five unit cells (with 3° bending magnets, U1, U2, U3, U4, U5) and two matching cells (with 1.5° bending magnets, M1, M2). Each of the unit and matching cells was machined from a single iron block (Johansson *et al.*, 2014 ▸). Additionally, all achromats contain two short straight sections (S1, S2) that host RF cavities, diagnostics, crotch absorbers (to pass synchrotron radiation to the front ends) and injection/diagnostic kicker magnets. Furthermore, long straight sections of ∼5 m (L) are available between each achromat and are reserved for insertion devices (with the exception of one straight used for injection). The layout of a standard achromat with the magnets and straight sections highlighted is presented in Fig. 2 ▸(*a*).

## 2. Design of the 3 GeV storage ring vacuum system   

In the 3 GeV storage ring, there are 97 sputter ion pumps installed. In one standard achromat four ion pumps are installed: in the long straight section (IP-L), in short straight section 1 (IP-S1), in short straight section 2 (IP-S2) and on the photon beam path going to the front end in unit cell 1 (IP-U1), as presented in Fig. 2 ▸(*b*). The cathodes used in the ion pumps are made of 50% tantalum and 50% titanium (noble diode) to pump noble gases more efficiently and stably. The majority of the ion pumps (77 units) have a nominal pumping speed of 75 l s^−1^; 20 units are of larger pumping speed as they are installed in the RF cavities and in-vacuum insertion devices.

### 2.1. Design of the vacuum chambers   

The low natural emittance required (328 pm rad – bare lattice) in a relatively small circumference was realized with a compact design for both the magnet apertures and the distance between magnets.

**Figure 3:**
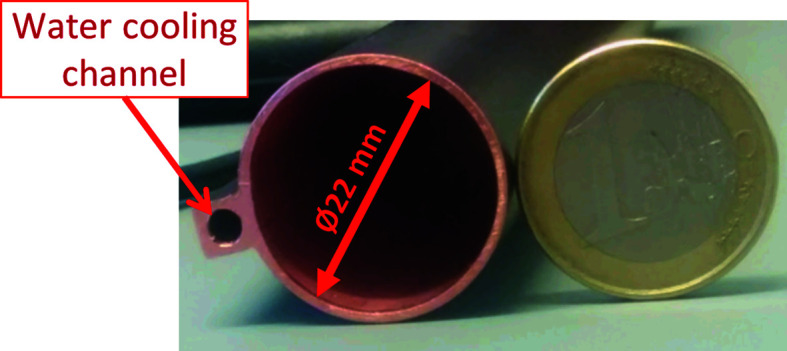
Cross section of a standard OFS vacuum chamber, with a welded cooling channel on the left-hand side and a 1 euro coin for size comparison on the right.

The magnets of the 3 GeV storage ring are small and have an aperture of 25 mm in diameter (Johansson *et al.*, 2014 ▸). The vacuum chambers inside the magnet blocks were designed with an internal diameter of 22 mm and a 1 mm wall thickness, leaving a clearance to the magnets of 0.5 mm. The vacuum chambers were made of oxygen-free silver-bearing (Ag 0.085%) copper (OFS-C10700) (Al-Dmour *et al.*, 2014 ▸). To cope with the heat from synchrotron radiation generated in the bending magnets, the vast majority of the chambers have a welded water cooling channel on one side, as shown in Fig. 3 ▸.

**Figure 4:**
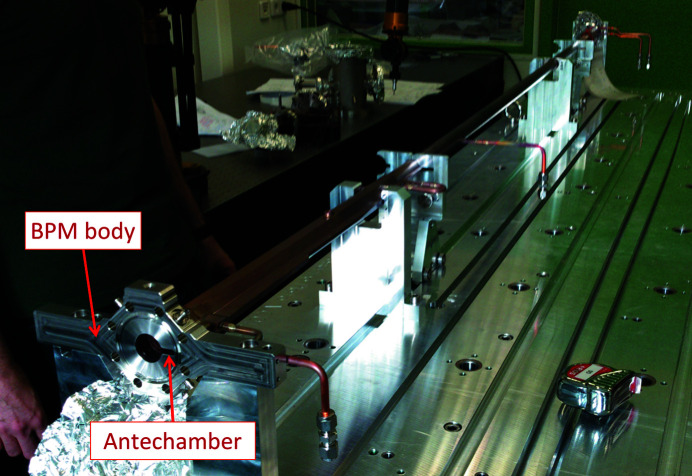
Dimensional check of a vacuum chamber (VC-1) using go–no-go movable tools, verifying the chamber outer geometry, with the BPM at the downstream end and an antechamber of 5 mm inner vertical aperture.

Most of the vacuum chambers do not have an antechamber. The only vacuum vessels with an antechamber are the ones where the synchrotron radiation is extracted to the beamlines – one section per achromat, around 1.3 m long. One vacuum chamber with a visible antechamber, at its downstream end (VC-1), is presented in Fig. 4 ▸.

Ten beam-positon monitors (BPMs) per achromat are installed and fastened directly onto the magnet blocks. Bellows with internal RF fingers are located on the extremities of the vacuum chambers (on each side of most of the BPMs). Their main purpose is to shield the BPMs from any deformation coming from the vacuum chambers, as well as to reduce the stress on the vacuum chambers due to heating from synchrotron radiation.

### 2.2. Non-evaporable getter (NEG) coating   

The NEG coating used at MAX IV is composed of Ti–Zr–V and was deposited using magnetron sputtering. It is characterized by a low photo-stimulated desorption (PSD) yield and a capability of pumping active gases – after activation under vacuum at a relatively low temperature of 180°C (Chiggiato & Kersevan, 2001 ▸; Benvenuti *et al.*, 2001 ▸). These two features make it a good candidate for vacuum systems of electron storage rings, where the total average dynamic pressure has to be in the range of 1 × 10^−9^ mbar (1 bar = 100 000 Pa) to ensure a long vacuum beam lifetime. NEG coatings are well incorporated in accelerators around the world (Hansson *et al.*, 2010 ▸; Herbeaux, 2008*b* ▸; Kersevan, 2002 ▸). They are especially beneficial when there is not enough space available for lumped pumps or absorbers and the vacuum chambers need to have a small inner diameter, which results in limited conductance. Similar constraints were one of the reasons why the MAX IV 3 GeV storage ring vacuum system was coated with an NEG film around 1 µm thick. In total, 98% of the 3 GeV storage ring circumference (lengthwise) was NEG coated. A few diagnostic elements (such as scraper blocks, striplines, stripline kickers, current transformer), the in-vacuum insertion devices and the RF cavities were not coated with NEG film. The ceramic pulsed magnet chambers were coated with titanium film.

In order to ensure that NEG coating by magnetron sputtering could be successfully applied to the MAX IV 3 GeV ring vacuum chambers, in 2011 a collaboration between CERN (European Organization for Nuclear Research, Geneva, Switzerland) and the MAX IV Laboratory was set up and an extensive research and development programme was accomplished (Costa Pinto, 2015 ▸; Grabski *et al.*, 2013 ▸).

The choice of NEG coating allows the simplification of the design of the vacuum system in several ways, *i.e.* reducing the number of lumped pumps, utilizing the chamber wall as a photon absorber, thereby reducing the number of required crotch absorbers, and removing the need for an antechamber to increase vacuum conductance. These benefits come with additional challenges related to manufacturing and also put stringent requirements on the substrate surface quality and cleanliness (Grabski *et al.*, 2013 ▸). Furthermore, special installation procedures must be followed and the use of equipment compatible with the NEG coating must be ensured. The NEG coating does not pump noble gases or methane. To mitigate this, ion pumps with cathodes designed to pump noble gases more effectively were used at MAX IV.

NEG films can be permanently contaminated when exposed to halogens (*e.g.* fluorine or chlorine) with no possibility of reactivation. Therefore, those substances must be kept away from the coated surfaces of the storage ring. Appropriate measures were taken to avoid halogens entering the vacuum system of the storage ring, such as the use of fluorine-free pumps and strict acceptance criteria of vacuum chambers for the storage ring and beamlines.

### 2.3. Production   

All the storage ring vacuum chambers [except ceramic kicker chambers, septum magnet, DCCT (direct current transformer) and RF cavities] were manufactured by a single company. This allowed good control of the whole production process, which had the following sequence:

(i) Surface treatment of extruded OFS copper tubes (performed at CERN).

(ii) Manufacturing of parts.

(iii) Assembly of vacuum chambers (welding, brazing).

(iv) Final cleaning of vacuum chambers.

(v) Welding of bellows assemblies at the chamber ends.

(vi) Dimensional and vacuum tests.

(vii) NEG coating.

The same company that manufactured the vacuum chambers also did the coating for 70% of the chambers. The remaining 30% were coated in collaboration with the ESRF (European Synchrotron Radiation Facility, Grenoble, France) and CERN (Grabski *et al.*, 2013 ▸). To monitor the quality of the NEG coating during production, all the vacuum chambers were visually inspected with an endoscope to identify possible uncoated areas and film non-conformities (peel offs). Furthermore, samples were coated along with the vacuum chambers and the surface chemical composition was evaluated with X-ray photoelectron spectroscopy (XPS). The film thickness was measured with a scanning electron microscope (SEM).

## 3. Testing and installation   

To activate the NEG coating all the vacuum chambers had to be heated (baked) under vacuum to at least 180°C, as described by Al-Dmour & Grabski (2017 ▸).

Test assembly and conditioning of one complete vacuum sector (achromat), excluding the long straight section, was performed off-site in the summer of 2014 with the first set of manufactured and NEG-coated vacuum chambers. A full set of vacuum chambers was installed on seven pre-aligned magnet blocks and supports. First the assembly was pumped down and leak tested. Next, the vacuum chambers were attached to a strongback and lifted to be baked in an oven following the procedure to be used during the final installation. This was done in order to validate the interfaces between the vacuum chambers and the magnets, and to establish and practise the installation procedure.

The on-site installation of the vacuum system of the 3 GeV storage ring started in November 2014 and finished in May 2015. The installation procedure for one achromat was as follows (Al-Dmour & Grabski, 2017 ▸):

(i) Assembly, pump down and testing of a full achromat on assembly tables, directly above the lower magnet blocks. The achromat was sealed with gate valves at the ends.

(ii) Connecting the vacuum achromat assembly (under vacuum) to a strongback and lifting up the achromat.

(iii) Baking oven installation around the achromat assembly.

(iv) Baking out (at 160°C) and activation (at 190°C) of the NEG coating inside a baking oven, with the chambers hanging from the strongback allowing the assembly to expand freely.

(v) Removal of the baking oven.

(vi) Leak testing and residual gas analyser spectra analyses.

(vii) Lowering the chambers to the assembly tables and adding components that could not be baked (epoxy clamps, BPM semi-rigid cables, corrector magnets).

(viii) Lifting up and removal of assembly tables and placement of the vacuum achromat on the lower magnet blocks.

(ix) Placement of the top magnet halves.

(x) Once two adjacent achromats were done, the straight in between was installed, baked and activated *in situ*.

The installation process described above took on average two weeks per achromat. There were two teams working in parallel for the installation of all 20 achromats. The RF cavities, injection straight, diagnostic components and insertion devices were baked *in situ*.

## 4. Vacuum commissioning and operation   

Each standard achromat of the 3 GeV storage ring has four ion pumps (three on the electron beam path and one on the photon beam path to the front end), one hot cathode extractor gauge (in S1) and one cold cathode gauge (in S2). The extractor gauges provide more accurate and stable readings, and their readings are not influenced by the photoelectrons that are generated by synchrotron radiation impinging on the vacuum chamber walls or absorbers. In addition, some achromats are equipped with a residual gas analyser (RGA) quadrupole mass spectrometer to evaluate the partial pressures. One of the RGAs is located in achromat 8-S1, where the photon and electron beams split. There the radiation fan between the beams is intercepted by an oxygen-free high conductivity (OFHC) copper crotch absorber which is not NEG coated. Another RGA is in achromat 17-L and is mounted where the electron beam path is fully NEG coated. With this instrumentation, it is possible to measure total and partial pressure around the storage ring, but they provide only localized measurements, as the chambers have low conductance.

The pressure measured inside the in-vacuum insertion devices is not part of any data presented in this work as it is not strongly dependent on the beam dose. Each in-vacuum insertion device is equipped with a large pumping capacity (several large NEG pumps and ion pumps). In the first two in-vacuum undulators (IVUs) installed in 2016, the total pressure with standard delivery beam current is currently below 2 × 10^−10^ mbar. The pressure in the remaining IVUs is less than 5 × 10^−10^ mbar.

The vacuum conditioning of the storage ring has progressed well and is evident from both the average pressure reduction and the increase in the total beam lifetime as the accumulated beam dose has increased.

As of October 2020, the storage ring had an accumulated beam dose of 3450 A h and the maximum stored beam current was 500 mA. Standard delivery to beamlines is at 250 mA with top-up every 10 min.

### 4.1. Average dynamic pressure rise   

The average base pressure (without beam) measured by the extractor gauges before the start of commissioning with electron beam was 2 × 10^−10^ mbar. The ion pumps provided an additional pressure indication of 8 × 10^−11^ mbar. In August 2015 the commissioning of the storage ring started. The first stored beam was on 15 September 2015, which resulted in a pressure increase to the high 10^−9^ mbar range.

**Figure 5:**
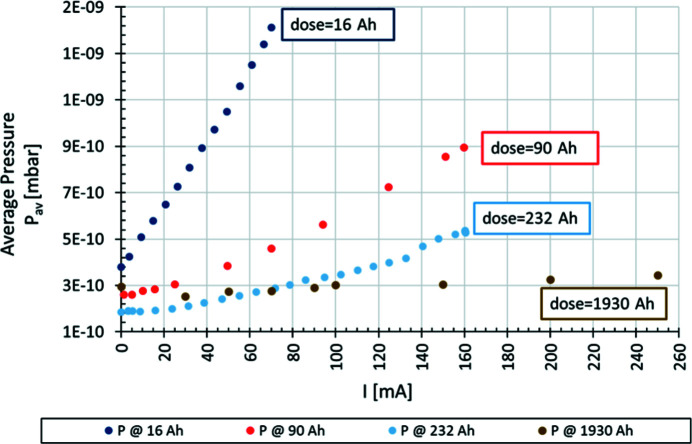
The average pressures (nitrogen equivalent) measured around the storage ring with extractor gauges in S1 versus current at various beam doses.

Fig. 5 ▸ presents the average pressure *P*_av_ (nitrogen equivalent), as measured by the extractor gauges installed around the storage ring, versus the beam current *I* at different accumulated beam doses. The measurements took into account extractor gauges installed in all of the achromats excluding several specific locations: achromat 1 (injection dipole kicker), achromats 9 and 15 (unreliable readings), and achromat 20 (diagnostic beamline, E1). The plot in Fig. 5 ▸ illustrates the decrease in measured pressure with increasing current, starting from the early commissioning stage (16 A h) to the later stages with a higher accumulated dose of 1930 A h.

**Figure 6:**
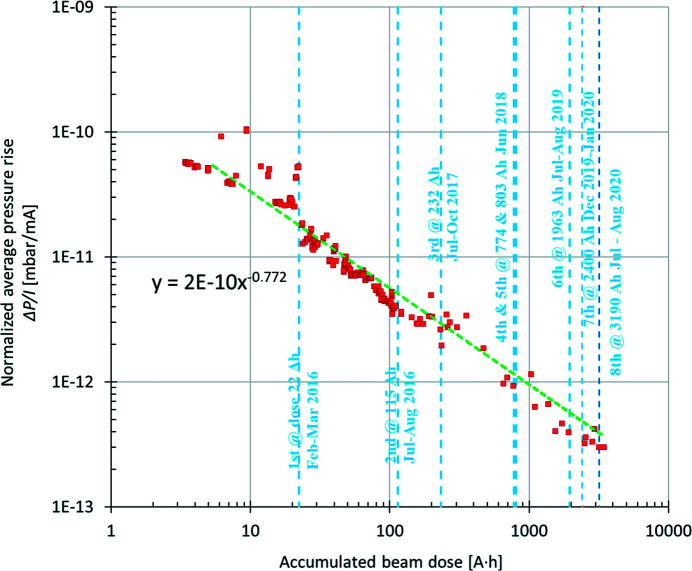
The normalized average pressure rise (mbar mA^−1^) versus the accumulated beam dose (A h). Blue vertical lines mark shutdowns, with the corresponding beam dose and date.

Fig. 6 ▸ shows the average pressure rise Δ*P*_av_ (from the total pressure with beam, the base pressure without beam was subtracted), as measured by extractor gauges (nitrogen equivalent), normalized to the beam current Δ*P*_av_/*I* (in units of mbar mA^−1^) as a function of the accumulated beam dose *x* (units of A h). The plotted data points were fitted to the following power function: *y* = 2 × 10^−10^ *x* ^−0.77^. From the fit it can be observed that the absolute value of the slope of the vacuum conditioning curve is 0.77. This value is slightly higher than those reported during the commissioning of other storage-ring-based synchrotron light facilities, meaning that the vacuum system of the MAX IV 3 GeV storage ring is conditioning faster. For example, at Diamond the absolute value of the slope was 0.7 (Cox *et al.*, 2008 ▸). After each shutdown, an increase in the normalized average pressure is usually observed. However, the average pressure recovers rapidly with further vacuum conditioning of around 5–30 A h, depending on the scale of the interventions performed. The higher pressure values following shutdowns were excluded from Fig. 6 ▸.

### 4.2. Beam lifetime   

**Figure 7:**
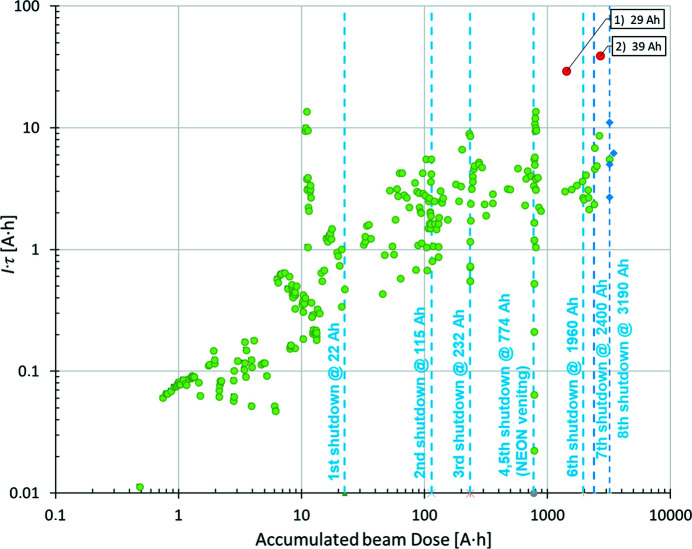
Normalized total beam lifetime *I*τ (A h) versus accumulated beam dose (A h). The two points marked in red are discussed in the text.

Fig. 7 ▸ presents the increase in the normalized total beam lifetime *I*τ (units of A h) versus accumulated beam dose (A h). The increase in the *I*τ product is an indication of the beam scrubbing effect, known as vacuum conditioning. The beam lifetime variations at a given level of accumulated dose are due to differences in beam size, bunch lengths and RF cavity settings rather than vacuum related. Typical total beam lifetime during delivery at 250 mA is 25 h.

The two points marked in red in Fig. 7 ▸ labelled 1) 29 A h and 2) 39 A h indicate the product of the current and beam lifetime measured when the beam was made intentionally unstable in the longitudinal plane by detuning the RF cavities at doses of 1430 and 2690 A h, respectively. By enlarging the beam longitudinally, the particle density in the bunches is reduced and the contribution of Touschek lifetime to the total lifetime is minimized. For both cases, the measurements were taken with a beam current of 350 mA, and total beam lifetimes of 83 and 111 h were measured, respectively, which can be considered lower limits for the vacuum-related beam lifetime.

Since the start of machine operation, there have been eight main accelerator shutdowns:

(i) At dose 22 A h, February to March 2016. Dedicated to the installation of two IVUs for the BioMax and NanoMax beamlines.

(ii) At dose 115 A h, July to August 2016. One in-vacuum wiggler (IVW) for the BALDER beamline and two NEG-coated chambers for elliptically polarized undulators (EPUs) for the HIPPIE and VERITAS beamlines were installed.

(iii) At dose 232 A h, July to October 2017. Prior to this shutdown no achromats were vented. This shutdown was dedicated to solving issues related to hot spots in the vacuum chambers and to the installation of a diagnostic beamline in achromat 2 (E5 in Fig. 1 ▸). Due to these activities, three achromats had to be vented and re-activated by baking (together with the corresponding long straight sections upstream and downstream of the vented achromat). Additionally, a multipole injection kicker (MIK) and a longitudinal kicker cavity (LK) were installed.

(iv) At dose 774 A h, June 2018. The neon venting technique was performed and validated as a way of avoiding re-activating the NEG coating by baking, which is usually required after an intervention that includes venting of an achromat. During this shutdown, two achromats were vented with purified neon.

(v) At dose 803 A h, July to August 2018. Devoted to the installation of one IVU for the CoSAXS beamline and one NEG-coated narrow-gap vacuum chamber for another EPU for the SoftiMax beamline (no achromats were vented). In addition, all installed 100 MHz RF cavities were vented with nitrogen (the nitrogen flow was maintained during the intervention) to adjust the cavities’ high power feedthrough coupling coefficient to better match the cavity for higher electron beam current operation. The cavities are equipped with gate valves upstream and downstream that were closed during the intervention. After the adjustment, the RF cavities were pumped down with turbo pumps without baking; the pressure recovered to acceptable vacuum level after 2 days.

(vi) At dose 1963 A h, July to August 2019. An upgraded version of the MIK chamber, an IVU for the DanMax beamline, a 100 MHz RF cavity and a current transformer were installed in straight sections. No achromats were vented.

(vii) At dose 2400 A h, December 2019 to January 2020. No vacuum interventions were performed in the 3 GeV storage ring.

(viii) At dose 3190 A h, July to August 2020. The shutdown included adding two new gate valves along the electron beam path to allow fast installation of a future RF cavity, and for this installation one achromat was vented. This was done utilizing the neon venting technique, similar to the one used during shutdown number (iv).

After each shutdown, an increase in the average pressure and a reduction in the total beam lifetime were observed. These changes were short-lived and recovery to previous values was quickly achieved.

### 4.3. Partial pressure measurement   

Given the fact that the RGA in achromat 17-L is mounted in an area where the electron beam path is fully NEG coated, it is believed that the spectra measured by this analyser reflect the gas composition in the majority of the storage ring circumference. The residual gas spectra listed in Table 1 ▸ were recorded with no stored beam and with beam current of 170–200 mA, for an accumulated beam dose of 450 and 705 A h.

The RGA measurements indicate mainly hydrogen (mass 2) in the gas spectrum. With 170 mA, there is an increase mostly in the amount of CO (mass 28). Methane (mass 16), water (mass 18) and CO_2_ (mass 44) are also observed but at similar levels as with no stored beam.

The listed partial pressures were obtained by dividing the values measured with the RGA (with sensitivity for nitrogen) by gas correction factors (ionization probability) corresponding to each gas species.

## 5. NEG coating performance   

Due to the scale of the NEG coating of the storage ring vacuum chambers, there was concern during the early stages of the project over the initial conditioning performance, long-term reliability, behaviour under repeated activations and the risk of saturation, and the resulting effects on machine operation and performance. However, from the early stages of commissioning it has been clear that the NEG coating has no negative influence on machine operation, and the vacuum performance was similar to or even better than that of the conventional vacuum systems used in other storage rings. In particular, a beam loss that could be attributed to the coating peeling off was never observed. During production and installation, extensive internal inspections were undertaken. Any chambers showing peel offs or coating damage were deemed unusable and were excluded from the installation.

During the production stage, after the chambers were NEG coated, around 15% of the chambers were activated at the supplier’s and collaborator’s premises. This was done to evaluate the coating activation behaviour and to check the sticking factor for hydrogen gas by performing pressure measurements. Similar sticking factor measurements were done at CERN on a few prototype chambers (Grabski *et al.*, 2013 ▸). In addition, to date, some of the achromats have been activated several times: two achromats were activated three times and six achromats were activated twice. The activation process (temperature and duration) was similar each time. The vacuum performance of those achromats (as measured by the vacuum gauges) is similar to that of the other achromats, which were activated only once. No signs of ageing of the NEG coating have been observed to date.

To evaluate the NEG coating performance, several tests were done with an electron beam circulating in the storage ring. The goal of the tests was to observe the beam and vacuum behaviour around the machine under different conditions.

### 5.1. Test with ion pumps off   

**Figure 8:**
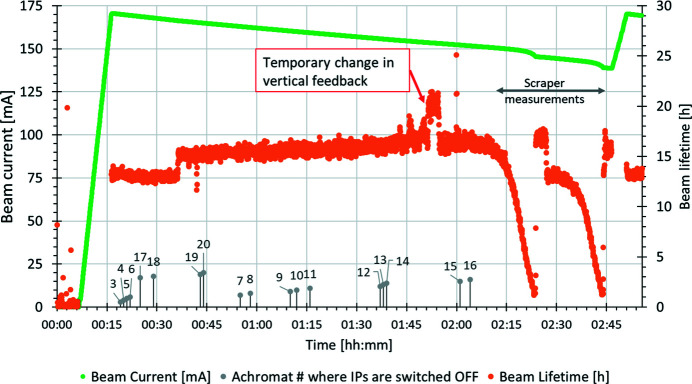
Beam current and lifetime during operation with the ion pumps switched off in the 3 GeV storage ring.

A test was performed with the majority of the ion pumps in the storage ring turned off to investigate possible saturation of the NEG coating. The test was done at an accumulated beam dose of 450 A h. During the test, an electron beam of 170 mA was injected into the machine (at time 00:16) with standard operating conditions, as presented in Fig. 8 ▸.

The total beam lifetime after injection was 13 h, resulting in a product of beam current and lifetime equal to 2.2 A h. The measured horizontal and vertical beam sizes were 22.2 µm and 9 µm, respectively.

During the test, 63 out of the 97 ion pumps installed in the storage ring were switched off. The ion pumps in the RF cavities (12 units), in-vacuum insertion devices (nine units), and diagnostic beamlines and injection straight (13 units) remained on during the test. The pressure was measured using the extractor gauges in short straight 1 (S-1) and cold cathode gauges in short straight 2 (S-2) in every achromat, along with two RGAs installed in achromats 8-S1 and 17-L. The beam current and lifetime during the 3 h period of the test are presented in Fig. 8 ▸. The grey dots with numbers mark the moments in time when the ion pumps of a particular achromat were turned off.

During the test, several scraper measurements were performed where the electron beam was scraped by movable copper collimators up to fraction of a millimetre from the beam centre. This was done to evaluate elastic, inelastic and Touschek scattering beam lifetimes. During the 2 h period, the total beam lifetime increased due to decay of the beam current, which is the expected behaviour. At 02:55 (almost 1 h of ring operation with 63 ion pumps switched off) the beam was topped up to 170 mA.

During the test, with the majority of the ion pumps switched off, no unusual decrease in the beam lifetime was observed which could be attributed to a pressure increase. The beam current and lifetime are summarized in Table 2 ▸. Furthermore, the radiation safety group performed measurements around the outside of the tunnel during the test and did not observe any increase in radiation levels above background.

Pressure measurements from the extractor and cold cathode gauges during the test are summarized in Table 3 ▸.

From Table 3 ▸ it can be observed that the pressure during operation with a beam current of 140–170 mA with ion pumps off increases by a factor of 3.3 and 3.8 (measured at locations S1 and S2, respectively) compared with the conditions when the ion pumps are switched on. Therefore, on average, the locally measured dynamic pressure is 3.6 times higher when operating with the majority of the ion pumps along the electron beam path switched off. The pressure stayed stable at these levels during the test.

#### 5.1.1. RGA spectra   

During the tests described above with the ion pumps switched off, RGA spectra were recorded at two locations in the ring, achromats 8-S1 and 17-L (long straight). Table 4 ▸ summarizes the amounts of the main gases (masses of 2, 16, 18, 28 and 44). To determine the contribution of each of the gas species to the total pressure, the RGA signal for each of the gases was divided by the corresponding gas correction factor. The remaining <1% for each case is attributed to other gas species.

The RGA positioned in 8-S1 is located in a chamber which is not NEG coated along the electron beam path (along a length of ∼20 cm). This chamber hosts the copper crotch absorber, which is also not NEG coated. The RGA which is installed in 17-L is located in an area which is fully NEG coated. This fact contributes to the difference in the gas species observed in the two RGAs.

#### 5.1.2. Beam dump test   

**Figure 9:**
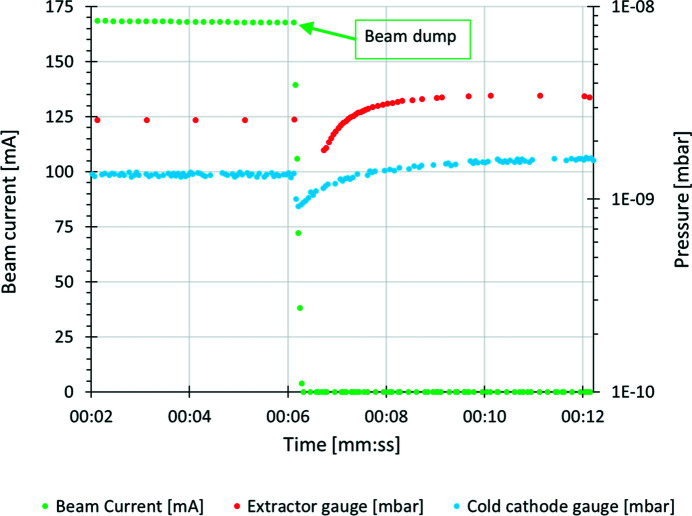
Beam current and pressures (N_2_ equivalent) measured during beam dump by the extractor gauge in S1 and cold cathode gauges in S2 in achromat 04 with the ion pumps switched off.

In another test run, the electron beam was dumped from 168 mA to 0 mA, with ion pumps switched off in achromats 3, 4, 5, 6 and 17. The pressures in achromat 4 were recorded by extractor and cold cathode gauges, located in S-1 and S-2, respectively, as shown in Fig. 9 ▸.

As presented in Fig. 9 ▸, the beam was dumped at time ∼00:06 and the total pressure decreased from a level of 2.6 × 10^−9^ (extractor gauge in S1) and 1.4 × 10^−9^ (cold cathode gauge in S2) mbar for around 1 min, then started to increase to a level of 3.5 × 10^−9^ and 1.6 × 10^−9^ mbar, respectively. This might be an indication of the effect of beam-induced pumping – the total pressure in this location (achromat 4-S1) without the circulating beam is higher than that with the beam (with the ion pumps turned off).

### 5.2. Comparison of simulated and measured pressure   

Monte Carlo simulations using *Synrad* and *Molflow+* (Kersevan *et al.*, 2020 ▸) were performed during the design phase to estimate the pressure profile at location S1 of an achromat. In that area a bare (not NEG coated) copper crotch absorber, an extractor gauge and an ion pump are installed. The chamber hosting the absorber is one of the few chambers that are not fully NEG coated: it is not coated for around 20 cm along the electron beam path but is NEG coated in the vertical direction. Upstream and downstream chambers are fully NEG coated. At that position, a high photon flux impinges the absorber, which results in high dynamic out­gassing. Therefore, the pressure profile and the possible effect of NEG film saturation for CO and CO_2_ were studied (Ady *et al.*, 2014 ▸).

A comparison of simulated and measured pressures at location S1 is presented in Table 5 ▸. At S1 locations in every achromat an extractor gauge is installed, so accurate pressure readings could be obtained without the influence of photoelectrons on the measurements.

The simulated total pressure in the S1 area at a beam dose of 100 A h and a 100 mA beam current was 1 × 10^−9^ mbar [Ady *et al.* (2014 ▸) reported a total pressure of 5 × 10^−9^ mbar for 500 mA]. The gas composition in the simulation resulting from the assumed outgassing data from bare copper, and the NEG coating saturation effect for CO and CO_2_, were taken into account.

The measured total pressure (including the base pressure of 1.9 × 10^−10^ mbar) at S1 locations in the 3 GeV storage ring at a dose of 100 A h with a beam current of 100 mA was 6.2 × 10^−10^ mbar (gauge reading, *i.e.* nitrogen equivalent). However, the measured partial pressure in the storage ring was hydrogen dominant (90% as listed in Table 4 ▸); accordingly, the hydrogen equivalent of the pressure reading was 1.3 × 10^−9^ mbar.

Although the simulated and measured total pressure values are very similar, the gas composition obtained from the simulations does not reflect what is measured at MAX IV. The difference might come from the limited NEG coating saturation effect for CO and CO_2_ and possible coating reactivation by the photon beam.

Future machines that have a vacuum system based on NEG-coated chambers may need to take into consideration that the dominating gas in such systems is hydrogen, which is the case at the MAX IV 3 GeV storage ring.

## 6. Major vacuum upgrades   

Several changes to the vacuum system following the initial installation in 2014–2015 have taken place and are summarized below:

(i) Insertion devices. Four in-vacuum undulators and one in-vacuum wiggler were installed. The interface to the machine vacuum system was done through cooled copper tapers and bellows on each side of the device. Moreover, three narrow-gap NEG-coated aluminium vacuum chambers of 8 mm internal vertical aperture and around 4.2 m length were installed together with EPUs.

(ii) Multipole injection kicker (MIK). In collaboration with the Soleil synchrotron, an MIK has been produced (Alexandre *et al.*, 2021 ▸) and its first version installed in the 3 GeV storage ring, achromat 2-L, in summer 2017 (see Fig. 1 ▸). The sapphire vacuum chamber is coated inside with a titanium thin film. Copper conductors are glued in eight grooves machined with very high precision. The MIK causes far fewer perturbations to the stored beam during injection compared with the standard dipole injection kicker (Dk) (Kallestrup *et al.*, 2019 ▸). In summer 2019 the second version of the MIK chamber (with higher manufacturing tolerances achieved and a thicker titanium coating) was installed in achromat 2-L. This upgrade reduced the perturbations of the stored beam by about two to three orders of magnitude compared with kicking the off-axis injected beam with the Dk that was used before.

(iii) Diagnostic beamlines (for emittance measurement). The 3 GeV storage ring has two diagnostic beamlines, E1 (achromat 20-M1) and E5 (achromat 02-U5) (see Fig. 1 ▸). The latter was not installed during initial installation in 2014–2015, due to a failure in the chamber production. During summer 2017 a new chamber was delivered and the E5 beamline was installed in achromat 02-U5.

(iv) Longitudinal cavity kicker (LK) (Olsson *et al.*, 2017 ▸). A pillbox cavity, with the inner cones made of copper brazed to the flanges of the steel chamber, was installed in achromat 11-S2 (see Fig. 1 ▸) in summer 2017.

(v) In summer 2020 two new gate valves, with a dummy chamber, were added in place of several vacuum chambers in the short straight section S2 of one of the achromats. This was done to reserve space for fast installation of a future RF cavity without the need to vent the entire achromat.

## 7. Installation and operational issues   

The two most challenging design constraints of the vacuum system of the 3 GeV ring were the small inner vacuum chamber apertures, with no place for lumped pumps and absorbers and the requirement for an NEG coating. These two aspects posed limitations on the installation and operation.

### 7.1. Installation issues   

An installation protocol was prepared and followed up for each installed achromat. As a result, the installation went smoothly and on schedule, with only minor issues to report.

#### 7.1.1. NEG coating   

**Figure 10:**
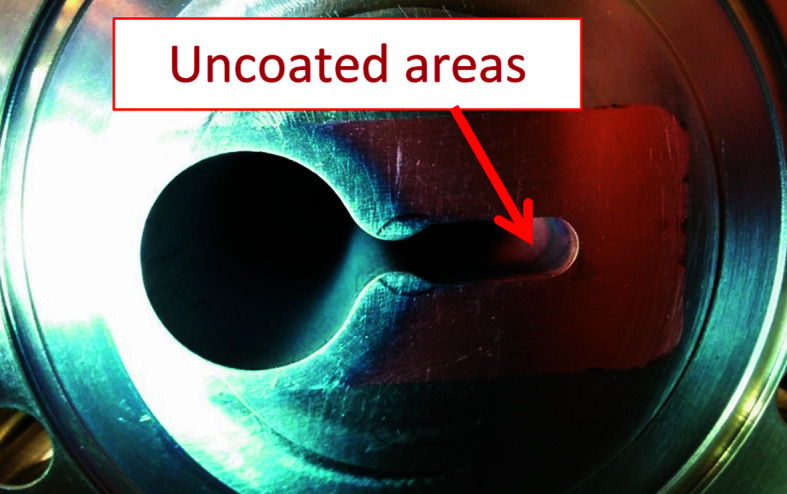
Uncoated areas of the antechamber with 5 mm vertical aperture.

**Figure 11:**
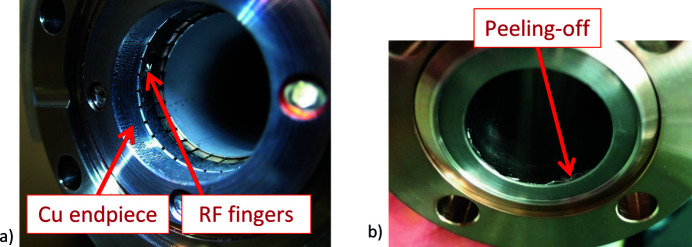
Visible peeling off of the NEG coating, (*a*) on an RF finger assembly and (*b*) on the edge of one of the chambers.

All the coated chambers were visually inspected before installation to check for non-conformities. The two main problems with the NEG coating were:

(i) Uncoated areas of the vacuum chambers. When the area of the uncoated part was small (less than a few square centimetres), the chamber was installed (see Fig. 10 ▸), otherwise the chamber was recoated, or else it was discarded and a new one was requested.

(ii) Peel off. The majority of the peel offs occurred in the RF finger assembly welded to the chamber ends. The RF fingers were requested to be shielded from the coating but were unnecessarily NEG coated [see Fig. 11 ▸(*a*)]. These RF finger and copper end-piece assemblies were replaced with newly manufactured RF fingers without an NEG coating. A few chambers had peel offs at the edges, and those chambers were discarded from the installation and replaced with spare units to continue installation [see Fig. 11 ▸(*b*)]. From around 650 NEG-coated vacuum chambers installed in the storage ring, only a few chambers were discarded during installation due to coating peel off that could not be remedied. The chambers that could not be installed due to coating issues constitute less than 1% of the total manufactured chambers. This issue could be avoided by thorough visual inspection of the chambers far ahead of installation.

#### 7.1.2. General issues   

**Figure 12:**
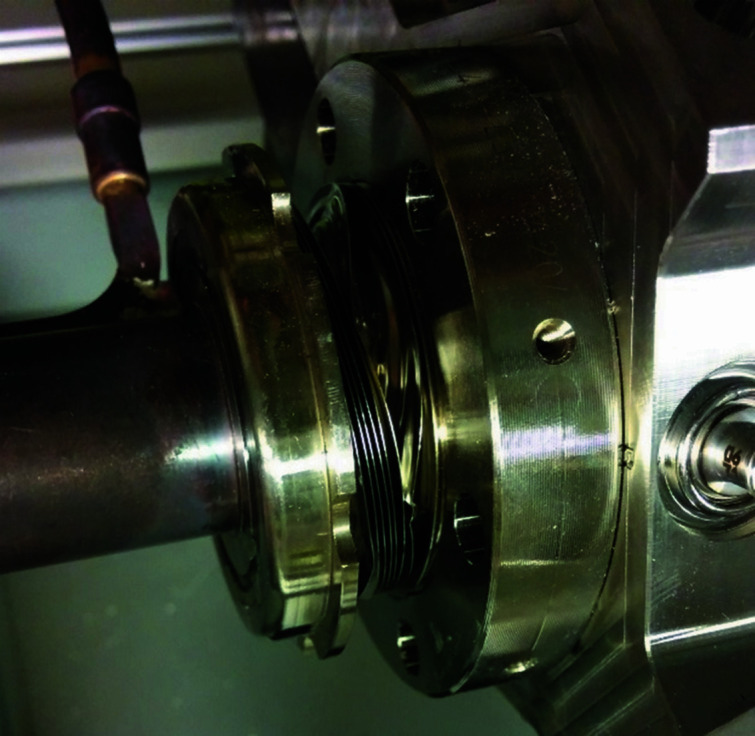
Deformation of an edge-welded bellows due to excessive torsion applied to the chamber end.

One edge-welded bellows was damaged during the manipulation (lifting/lowering) of the achromat. Excessive torsion was applied to the chamber and the convolutions of the bellows at one end were deformed (see Fig. 12 ▸).

One already installed achromat was accidentally vented with atmospheric air during machine protection system tests, before machine startup. The full vacuum procedure, including baking and NEG coating activation of this achromat, needed to be repeated.

### 7.2. Operational issues   

During 2017 there was one vacuum failure, contributing 0.3% of the total downtime of the 3 GeV storage ring that year. In 2018 there were eight vacuum alarms resulting in beam dumps, contributing 2.4% of the total machine downtime. In 2019 and 2020, the storage ring had, respectively, uptime of over 97% and 97.4% of scheduled time for delivery to beamlines, while vacuum-related downtimes were, respectively, 1.2% and 2.7% of the total downtime. The main contributor to downtimes due to vacuum is from alarms triggered by ion pumps or vacuum gauges when a measured pressure reaches the upper defined pressure limit and results in a beam dump and closure of gate valves. Those pressure spikes are short lived (several seconds) but are high enough to trip the vacuum interlock and dump the beam.

**Figure 13:**
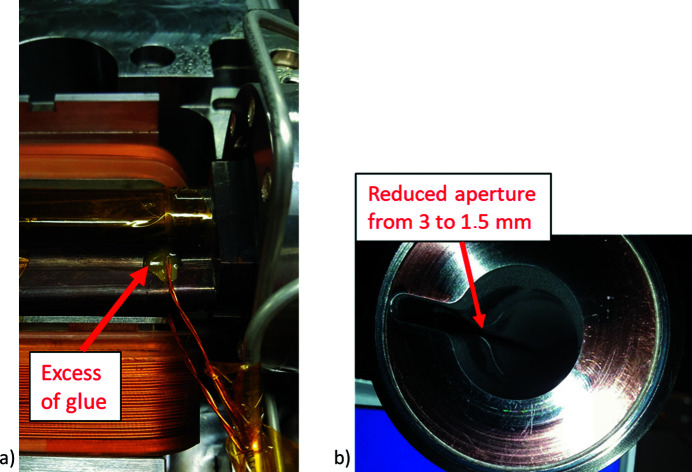
(*a*) An excess of glue applied to a vacuum chamber exterior. (*b*) The reduced inner aperture of the chamber due to an excess of glue.

A few limitations were encountered during machine operation. In the 3 GeV storage ring, the vacuum chambers directly intercept the dipole synchrotron radiation, so the temperature of the vacuum chambers is monitored by thermo­couples glued on the vacuum chambers in critical areas (around 30 thermocouples per achromat). On several chambers, the recorded temperature level during operation was higher than simulated. A few hot spots were identified and investigated. The causes of such problems are summarized below:

(i) Positioning of the vacuum chambers. Some vacuum chambers (in straight sections L, S1, S2) were not positioned correctly due to geometric non-conformity or deformation. Consequently, the beam was striking areas that are not designed to intercept the beam. Once alignment of those was performed, the hot spots were eliminated.

(ii) Chamber non-conformities. After installation, some crotch absorbers (installed in S1) did not shield the radiation fan in the correct spot. The straightness of the chambers and some tolerances did not meet the technical specifications defined on the drawings. In some cases, the absorbers were exchanged for longer ones, and in other cases, finite element analysis was performed to check if the chambers could tolerate the additional heat load. If the non-conformance was found to be safe, no further action was taken.

(iii) Deformed chambers during installation. Thermocouples were glued with epoxy in selected locations that were considered critical. On a few vacuum chambers, a thermocouple placed in the vicinity of the photon beam extraction was mis-positioned and glued with an excessive amount of epoxy, as illustrated in Fig. 13 ▸(*a*). When the magnet in that location was closed, it pressed the chamber through the glued thermocouple and caused deformation of the chamber. As a result, the photon beam was heating up the deformed area which is shown in Fig. 13 ▸(*b*). This imposed a limitation on the beam current and the use of one insertion device where the photon beam could cause excessive heating. These problem­atic chambers were exchanged in three achromats during the third shutdown in 2017. After the chambers had been exchanged, the achromats were baked and the NEG coating was re-activated, and this action resolved the issue.

## 8. Neon venting   

Up until spring 2018, the procedure that was followed for any intervention related to venting of vacuum chambers in the 3 GeV storage ring, that are located within an achromat, was similar to that during installation:

(i) Vent the achromat and the two adjacent straight sections and disconnect the two straight sections.

(ii) Open all top magnet halves.

(iii) Connect the vacuum chambers to the strongback and raise all the vacuum chambers.

(iv) Install assembly tables over the lower magnet blocks and lower the achromat onto the assembly tables.

(v) Exchange the faulty chamber, pump down and leak test.

(vi) Raise the achromat, install the oven, and perform the bakeout and activation of the NEG coating.

(vii) Remove the oven and lower the chambers onto the assembly tables, and install non-bakable components to the vacuum chambers.

(viii) Raise the achromat again and remove the assembly tables.

(ix) Lower the achromat back onto the lower magnet blocks.

(x) Close the magnet blocks, reconnect all the water and electrical systems and check alignment.

(xi) Connect upstream and downstream long straight sections to the achromat, and bake and activate *in situ* the NEG coating in the long straight sections.

For one achromat, this procedure would take two weeks, and up to four weeks in cases where the insertion devices were installed in the adjacent long straight sections. Such a procedure is lengthy and includes risks during the manipulation of the chambers and the opening of the magnets.

MAX IV looked at other alternatives which could reduce the time of intervention, reduce the risk and simplify the procedure. This could be achieved by venting the vacuum system using ultra-pure neon gas, therefore limiting the saturation of the NEG film during the intervention and thus avoiding the need to bake and re-activate the coating.

The neon venting procedure was used for activated NEG-coated vacuum chambers at CERN for interventions in the LHC long straight sections and detector chambers. The main idea behind the procedure is to avoid saturating the NEG coating during the intervention. Neon was used as it is an inert (noble) gas, thus is not pumped by the NEG coating, it has relatively low atomic mass (interacts less with the electron beam) and does not affect leak detection (for leak detection helium and argon are used). During such intervention, ultra-pure neon is used for venting and is kept at slightly above atmospheric pressure inside the chamber. When the chamber to be exchanged is removed, the flow of neon is sustained, which prevents atmospheric air from back streaming to the inside and saturating the NEG coating, thus eliminating the need for baking and re-activation of the film (Bregliozzi, 2009 ▸; Lanza *et al.*, 2011 ▸).

Following discussions with CERN, two neon venting stations were built at MAX IV. In addition, a test stand was set up consisting of 16 m of NEG-coated vacuum chambers, which were activated and then vented with neon gas, and then the vacuum performance was evaluated. The main objective of the test was to get familiar with the use of the neon stations before using them for machine intervention, and to investigate the effect on vacuum recovery after venting.

Following the successful results, a scheduled machine intervention took place in June 2018. The scope was to exchange two faulty vacuum chambers (as presented in Fig. 13 ▸) and one crotch absorber (all parts located in S1) which required two achromats to be vented. The intervention with injection of neon and the actual replacement of faulty components took around 30 min per achromat. The installed components were stored in air at atmospheric pressure before the intervention. The pump down with turbomolecular pumps and ion pumps took 5 days. The activation of the NEG was not performed during this process. Following the intervention, dedicated machine time was scheduled with the main purpose of evaluating the effect of the performed intervention on the machine performance.

### 8.1. Pressure around the storage ring post neon venting   

After the intervention, the pressure reading from the extractor gauges around the ring did not change in comparison with before the intervention, with the exception of the gauges located in the same achromats where the exchanges were done (S1). There the reading was two orders of magnitude higher, but recovered to the value before the intervention after around 50 A h of beam conditioning.

**Figure 14:**
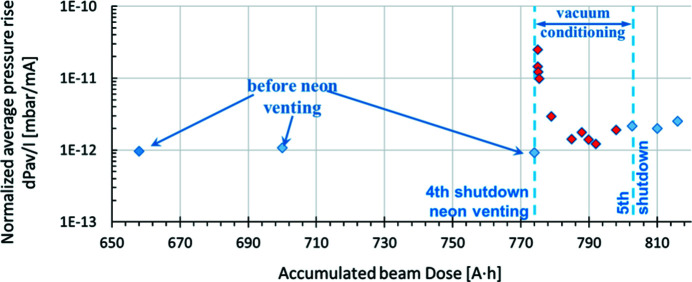
The normalized average pressure rise versus accumulated beam dose before and after the first neon venting in 2018.

The normalized average pressure rise before the inter­vention was 9.3 × 10^−13^ mbar mA^−1^. Following the inter­vention and after 1 A h of operation, this value increased to 2.4 × 10^−11^ mbar mA^−1^. However after 18 A h of beam conditioning it reduced to 1.2 × 10^−12^ mbar mA^−1^, as presented in Fig. 14 ▸. In comparison, during the 2017 machine intervention, in which neon venting was not used and three achromats were vented and reactivated in the standard way, 40 A h of accumulated dose was required to recover the average pressure in the storage ring.

### 8.2. Beam lifetime post neon venting   

**Figure 15:**
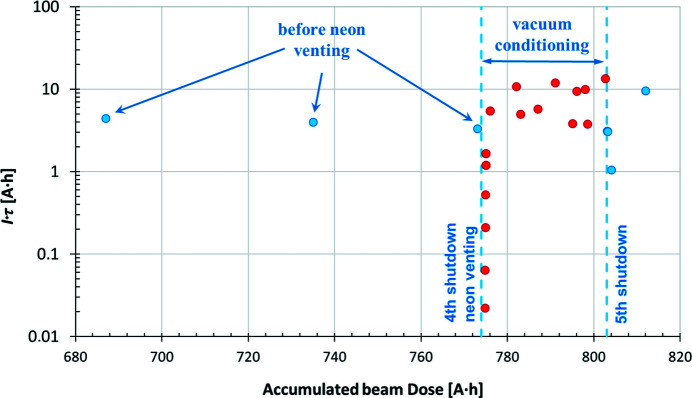
3 GeV storage ring: the normalized lifetime *I*τ (A h) versus accumulated dose (A h) before and after the neon venting in 2018.

The total beam lifetime before the intervention was between 3 and 5 A h. Just after the intervention (after a dose of 1 A h), this value reduced to 1.2 A h, and during the first 10 A h of beam conditioning the lifetime increased back to the original value from before intervention (3.8 A h), as shown in Fig. 15 ▸.

### 8.3. Partial pressures measurements post neon venting   

Two new RGAs were installed without bakeout in the places where the chambers were replaced. After the inter­vention, and without the electron beam, neon peaks (mass 20 and 22) were evident in the scans at those two locations. With the first stored beam, the partial pressure of neon gas increased by one order of magnitude close to the intervention areas. After 18 A h of beam conditioning, the neon partial pressures dropped by one order of magnitude (with beam) without any limitation on the accelerator operation and beam delivery to the users. No neon peaks were observed in the other RGAs around the machine, with or without stored beam.

### 8.4. Adding two gate valves   

During the eighth shutdown in summer 2020, two new gate valves were installed in short straight section S2 of one of the achromats in place of straight vacuum chambers, reserving space for future fast installation of an RF cavity. In order to add the two new gate valves, the achromat needed to be vented and a few chambers replaced. To reduce the inter­vention time a similar neon venting procedure to the one used in 2018 was developed, although in this intervention longer chambers were replaced than in the previous intervention of 2018. Both of the chambers to be installed had gate valves preinstalled at one end. The chambers were baked and the NEG coating was activated before installation. This approach was chosen to reduce the saturation of the NEG coating of the installed chambers to a minimum. The intervention procedure was rehearsed several times on a mock setup before the shutdown to ensure smooth execution. The final procedure for the intervention was as follows:

(i) Two short NEG-coated vacuum chambers were prepared. One gate valve was mounted on each chamber and the all-metal neon venting system was connected with a flexible hose. Both chambers were baked and the NEG coating was activated. These chambers were kept under vacuum, together with the attached neon venting system, until the installation time.

(ii) Prior to the intervention two neon stands were pumped down and their NEG filters were conditioned. The NEG filters are part of the neon venting system and used to purify the injected neon gas from unwanted residues that it may contain.

(iii) Two days before the intervention, the two neon stands were connected through right-angle valves to short straight section S1 of the achromat to be modified and long straight section L of the next achromat. The two connection places were pumped down and baked with two additional pumping stations.

(iv) On intervention day the achromat was vented with neon from both sides: S1 (upstream S2) and L of the next achromat (downstream S2). The neon flow was sustained while the vacuum chambers were removed from short straight section S2.

(v) The new upstream chamber with a gate valve was vented with neon from the gate valve side and a flow of neon was established. After the chamber had been installed on the upstream side, the new gate valve was closed and the neon flow from the upstream achromat side was stopped. The upstream part of the achromat was pumped down from short straight section S1.

(vi) The new downstream chamber with a gate valve was vented with neon from the gate valve side and a flow of neon was established. After the chamber had been installed on the downstream side, the new gate valve was closed and the neon flow from the downstream achromat side was stopped. The downstream part of the achromat was pumped down from long straight section L of the next achromat.

(vii) After approximately 24 h of pumping, the pressure measured by the extractor gauge in the upstream side (S1) was 1 × 10^−9^ mbar and the ion pumps were switched on.

(viii) After 4 d, the pumping stations were disconnected and the right-angle valves closed, and the pressure at measurement location S1 was 4 × 10^−10^ mbar, which was around 10% higher than before the intervention.

(ix) In the place between the two newly installed gate valves (S2), a dummy straight vacuum chamber was installed with tapers and an ion pump. It was pumped down and baked and the NEG coating was activated *in situ* following the standard procedure.

The time from the start of the venting of the achromat until the start of the pumping down with the newly installed chambers and gate valves was approximately 1 h.

After the shutdown had finished, the beam lifetime was recovered to a nominal value of 5 A h with a beam dose of several A h. The intervention did not have any negative impact on the operation of the storage ring. This can be observed in Figs. 6 ▸ and 7 ▸, where the last points were measured after the eighth shutdown had finished.

## 9. Conclusions   

The vacuum system of the 3 GeV storage ring of MAX IV is unique, considering the small aperture of the vacuum chambers, the extent of the applied NEG coating and the low number of ion pumps and crotch absorbers.

The vacuum conditioning has progressed well with the accumulated beam dose, and the electron beam lifetime continues to increase as the average pressure around the ring continues to decrease. The slope (absolute value) of the vacuum conditioning curve presented in Fig. 6 ▸ is 0.77 (*x* ^−0.77^, where *x* is the accumulated beam dose) and this is slightly better than what has been observed in other synchrotron light facilities, 0.7 (Cox *et al.*, 2008 ▸) and 0.69 (Herbeaux, 2008*a* ▸).

There were no operational issues related to the NEG coating, which shows this technology to be reliable and effective in ensuring low dynamic pressure. Initial concerns with the risk of saturation of the NEG in the early stages of commissioning when pressures are high has proven not to be an issue. The same is true regarding the risk of the coating peeling off and affecting the beam lifetime. Finally, after five years of operation and several interventions including reactiv­ations in a number of achromats, the long-term robust performance of a nearly 100% NEG-coated accelerator vacuum system is well established.

A few issues with hot spots on the vacuum chambers were the main obstacles encountered during vacuum system conditioning. However, the impact on machine operation was minor.

The maximum design current of 500 mA was achieved in November 2018 and the total beam lifetime reached 5 A h after an accumulated dose of approximately 100 A h.

The use of neon venting (on two occasions in 2018 and 2020) as the procedure to be followed for vacuum inter­ventions that involve replacing vacuum chambers and components significantly reduced the intervention time and possible risks associated with splitting magnets and reactiv­ating the NEG coating. This procedure has a negligible effect on the machine performance, and proved to be efficient and robust.

## Figures and Tables

**Table 1:** Selected partial pressures as measured by the RGA in achromat 17-L at accumulated beam doses of 450 and 705 A h at no stored beam and at 170 and 200 mA stored beam

			Mass (gas species)				
RGA location	Beam current (mA)	Accumulated beam dose (A h)	2 (H_2_)	16 (CH_4_)	18 (H_2_O)	28 (CO)	44 (CO_2_)
17-L	0	450	98.7%	0.2%	0.1%	0.8%	0.1%
	170	450	94.7%	0.4%	<0.1%	4.2%	0.3%
	0	705	98.9%	0.2%	<0.1%	0.8%	0.1%
	200	705	95.5%	0.3%	<0.1%	3.9%	0.1%

**Table 2:** Summary of beam current (*I*), lifetime (τ) and product *I*τ during beam operation with most of the ion pumps (IP) switched off

Time from injection (hh:mm)	Current (mA)	Lifetime (h)	*I*τ (A h)	Comment
00:16	170	13	2.21	Before the start of the test (all IP are on)
02:07	150	16	2.40	All IP are off†
02:45	139	15.5	2.15	After scraper measurement and all IP are off†
02:46	170	13	2.21	Beam current top up and all IP are off†

† Except ion pumps in RF section, injection straight and insertion devices.

**Table 3:** Average pressures from achromats around the 3 GeV storage ring measured in sections S1 (extractor gauges) and S2 (cold cathode gauges) with no beam, and with circulating beam 140–170 mA with sputter ion pumps on and off

Beam current (mA)	Ion pumps status	S1 (extractor gauges) average pressure (mbar)	S2 (cold cathode gauge) average pressure (mbar)
0 (base pressure)	On	2.7 × 10^−10^	2.3 × 10^−10^
140–170	On	4.1 × 10^−10^	4.5 × 10^−10^
140–170	Off	1.4 × 10^−9^	1.7 × 10^−9^
	Pressure ratio (with beam)	3.3	3.8
	Average pressure ratio	3.6	

**Table 4:** Partial pressures measured with the RGAs in achromats 8-S1 and 17-L with no stored beam, with stored beam at standard operation (ion pumps on) and with stored beam with ion pumps off

				Mass (gas species)				
RGA location	Beam current (mA)	Ion pump status	Accumulated beam dose (A h)	2 (H_2_)	16 (CH_4_)	18 (H_2_O)	28 (CO)	44 (CO_2_)
8-S1	0	On	450	97.9%	0.4%	0.1%	1.3%	<0.1%
	163	On		90.2%	0.8%	<0.1%	7.7%	0.2%
	146	Off		73.4%	6.3%	0.1%	16.1%	0.1%
17-L	0	On		98.7%	0.2%	0.1%	0.8%	0.1%
	170	On		94.7%	0.4%	<0.1%	4.2%	0.3%
	140	Off		95.7%	1.2%	<0.1%	2.8%	0.1%

**Table 5:** Comparison of simulated and measured total pressures and gas compositions at S1 locations at a dose of 100 A h and a beam current of 100 mA

				Mass (gas species)				
	Beam current (mA)	Accumulated beam dose (A h)	Pressure (mbar)	2 (H_2_)	16 (CH_4_)	18 (H_2_O)	28 (CO)	44 (CO_2_)
Simulated	100	100	1 × 10^−9^	16%	2%	0%	36%	46%
Measured	100	100	1.3 × 10^−9^ (H_2_ equivalent)	90%	0.8%	<0.1%	7.7%	0.2%
